# The prescription talk – an approach to teach patient-physician conversation about drug prescription to medical students

**DOI:** 10.3205/zma001095

**Published:** 2017-05-15

**Authors:** Katarina Hauser, Armin Koerfer, Mathilde Niehaus, Christian Albus, Stefan Herzig, Jan Matthes

**Affiliations:** 1 Universität zu Köln, Zentrum für Pharmakologie, Institut II, Köln, Deutschland; 2 Uniklinik Köln, Klinik und Poliklinik für Psychosomatik und Psychotherapie, Köln, Deutschland; 3 Universität zu Köln, Humanwissenschaftliche Fakultät, Lehrstuhl für Arbeit und Berufliche Rehabilitation, Köln, Deutschland; 4 Universität zu Köln, Rektorat, Köln, Deutschland

**Keywords:** prescribing, drug information, medical education, clinical pharmacology, patient-physician relation, communication, simulation

## Abstract

**Background:** Medication communication from physicians to patients often is poor, by this among others enhancing the risk of non-adherence. In this context, a neglect regarding the prescription talk has been complained.

**Aim of the project: **In a newly developed elective medical students work on physician-patient conversations dealing with drug prescription. Essential aspects related to an effective and safe drug treatment are combined with steps of shared decision-making. Together with a tutor, students develop a (model) conversation guide that might be tailored according to individual needs and views.

**Description/Methods: **In a one-week course 3rd-5th year medical students treat a paper case according to problem-based learning. This is accompanied by a one-hour lecture and literature provided on an online learning platform (ILIAS). During a workshop, aspects of drug treatment and patient participation are integrated into a guide for a prescription talk. At the end of the week the students are invited to apply the (if need be individualized) guide in a simulated physician-patient communication with an actor. The conversation is evaluated using a checklist based upon the (model) conversation guide.

**Results: **Informal and formalized feedback indicate high acceptance and satisfaction of participants with this elective. The checklist turned out to be of acceptable to good reliability with mostly selective items. Portfolio entries and written evaluation suggest that participants’ positions and attitudes are influenced.

## Zusammenfassung

**Hintergrund: **Die Arzt-Patient-Kommunikation über die Arzneitherapie zeigt häufig Defizite, was u.a. eine unzureichende medikamentöse Adhärenz zur Folge haben kann. Vernachlässigt wurde in diesem Zusammenhang lange Zeit das Verordnungsgespräch.

**Zielsetzung: **Im neu konzipierten Wahlpflichtblock setzen sich Studierende der Humanmedizin aktiv mit Arzt-Patient-Gesprächen im Rahmen der Arzneiverordnung auseinander. Berücksichtigung finden wesentliche Aspekte einer sicheren und effektiven Arzneitherapie sowie das Modell der Partizipativen Entscheidungsfindung. Unter Anleitung entwickeln die Studierenden einen Gesprächsleitfaden, dessen Anwendung auch individuellen Bedürfnissen und Vorstellungen Rechnung tragen soll.

**Beschreibung/Methoden: **In diesem einwöchigen Kurs erarbeiten sich die Studierenden wichtige Hintergrundinformationen anhand eines Fallbeispiels im Rahmen des Problem-basierten Lernens. Unterstützt werden sie durch ein Impulsreferat und auf einer elektronischen Lernplattform angebotene Literatur. Die gemeinsame Synthese der Aspekte zu Arzneitherapie und Patientenbeteiligung in Form eines Gesprächsleitfadens erfolgt in einem interaktiven Workshop. Am Ende der Woche können die Studierenden sich und den (Muster-) Leitfaden in einem mit einem Schauspielerpatienten simulierten Verordnungsgespräch ausprobieren, das anhand einer Checkliste beurteilt wird.

**Ergebnisse: **Informelle Rückmeldungen und die standardisierte fakultätsweite elektronische Evaluation zeigen ein hohe Akzeptanz und große Zufriedenheit seitens der Studierenden. Die Checkliste erlaubt eine weitgehend reliable Beurteilung der Gespräche anhand überwiegend trennscharfer Items. Portfolioeinträge und Freitextevaluationen sprechen dafür, dass bei den Teilnehmern Einstellungen und Haltungen beeinflusst werden.

## 1. Introduction

During every second physician-patient consultation a drug is prescribed [1], [2]. All over the world poor (medication) adherence (i.e. non-adherence) is an essential problem [3]. Sufficient adherence on the other hand can improve treatment results [e.g. [4], [5]. Non-adherence is often due to poor communication between physicians and patients. Furthermore, treatment decisions are often made by the physician [e.g. [6], [7], though patients want to participate [e.g. [8], [9].

Findings that in only about one third of prescription talks physicians touch risks and adverse events [10], [11], though these issues are of particular importance from the patients’ view [12], [13], indicate essential deficits in medication communication. One reason for physicians’ communication deficits is that this subject has been largely neglected in under- and post-graduate education [14], [15]. However, particularly for young doctors the ability to communicate in a patient-oriented manner and to inquire important information about the patient is an essential, “non-pharmacological” prerequisite for safe drug prescribing [16].

Here we introduce a project aiming at the improvement of physicians’ key skills. The project is part of a Cologne curriculum on (medical) communication and emphasizes enhancement of (medication) adherence via purposeful communication and shared decision-making (SDM). A (model) guide is developed to help medical students coping with subject- and communication-related requirements by conducting a structured medication conversation. Students are given the opportunity to practice their communication competencies in a simulated prescription talk. A further aim was to force students to critically deal with potentials and chances of communication in this particular medical field and by this to sensitize students and to influence their attitudes.

A checklist developed along our (model) conversation guide was used to evaluate simulated prescription talks.

## 2. Description of the project (methods)

### 2.1. Setting

In the Cologne medical curriculum, electives are to deepen contents of disciplines and areas that are of a student’s particular interest. Since the winter term 2013/2014 the Centre of Pharmacology offers the one-week elective described here. Students who have passed the course on basic pharmacology are allowed to attend. The extent of 8-9 hours in total is similar to other electives offered at our medical faculty. Maximum number of participants is restricted to 12 considering the teaching formats. The course was conducted by KH and JM.

#### 2.2. Contents and (teaching-) methods

##### 2.2.1. Units and process of the course

**Figure 1:**
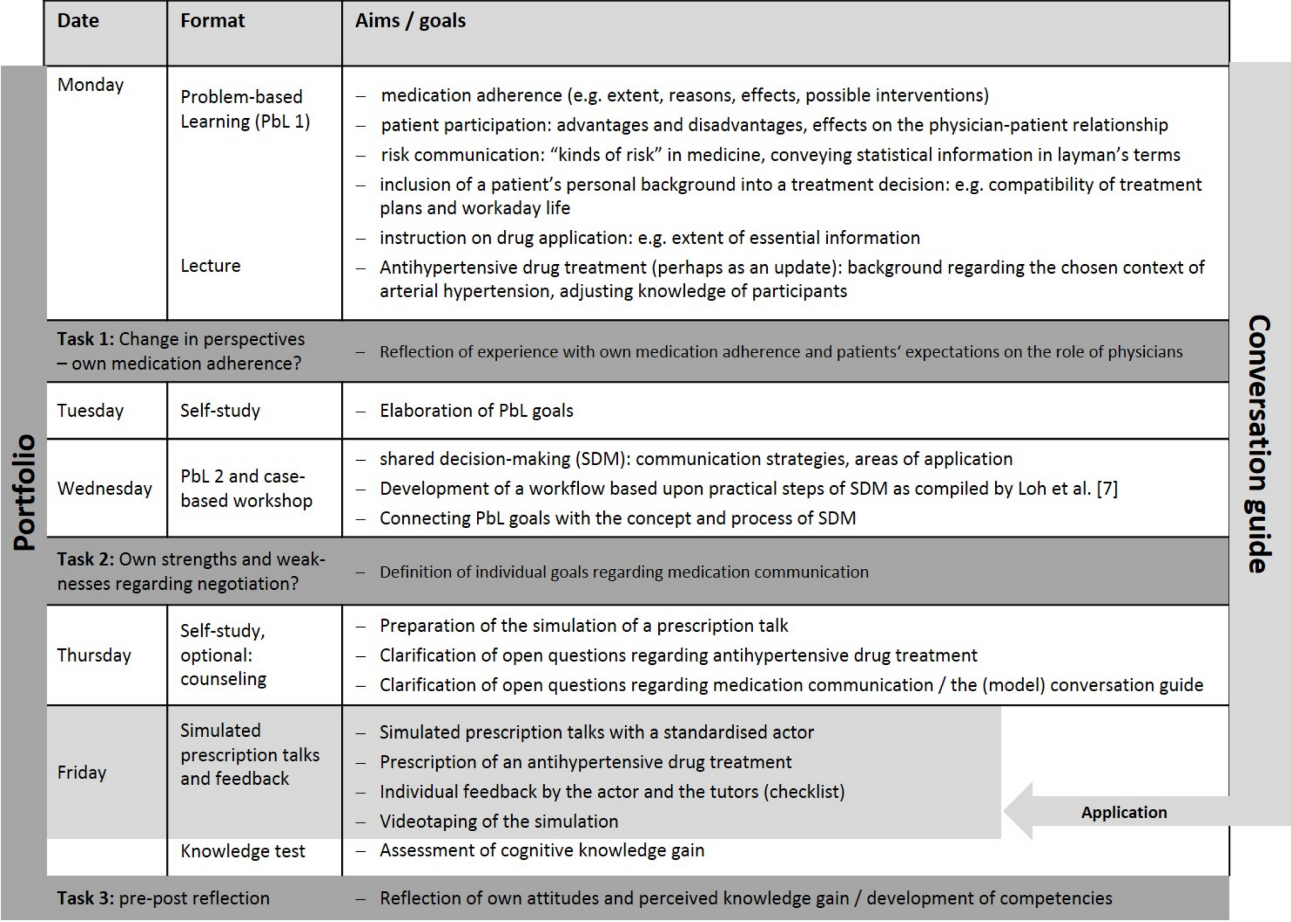
Synopsis of the time schedule, learning / teaching formats and contents of our elective on medication communication.

Traditional and more innovative methods are combined. Using the example of arterial hypertension paper cases are provided to prompt students to work out and deepen contents related to drug prescribing and medication communication. The following aspects were considered to be relevant regarding a prescription talk (see Figure 1 (Fig. 1)):

- medication adherence (e.g. extent, reasons, effects, possible interventions)
- shared decision-making (e.g. steps and strategies, areas of application, effects on physician-patient relationship)
- risk communication (e.g. “kinds of risk” in medicine, conveying statistical information in layman’s terms)
- inclusion of a patient’s personal background into a treatment decision (e.g. compatibility of treatment plans and workaday life)
- instruction on drug application (e.g. extent of essential information).

The elective starts with the participants treating a paper case according to problem-based learning (PbL). In brief, the case describes a suboptimal physician-patient communication in the context of a first-time prescription of an antihypertensive drug. The patient is insecure regarding how to take his medication, the drug’s effects and side effects and in the end becomes non-adherent. At the end of the first PbL session of about 45 minutes students define learning goals that – due to case construction – should be related to the relevant aspects mentioned above. Immediately after that a short input on basics of antihypertensive drug treatment is given. Two days later students meet for the second PbL session to discuss what they have found regarding their learning goals. For treating learning goals, some relevant publications are provided by an internet-based learning platform (ILIAS). The second PbL session is immediately followed by a workshop where the students together with a staff tutor develop a guide for a medication conversation based upon the aspects of drug treatment and patient participation discussed so far. Since in some settings shared decision-making (SDM) has been shown to be outcome-relevant [17], [18], SDM criteria as described by Charles et al. [19], [20] and the derived practical steps [7] are considered. Furthermore, recommendations on how to give essential drug information are addressed [11]. The overall aim is to develop a guide that acts as an aid to orientation regarding coverage of important contents and giving a helpful structure for a prescription talk. The students have to decide by themselves whether and to what extent they follow this guide in the coming simulation, since we do not want to demand too much of them in that short time period. Furthermore, an important issue of communication is authenticity and this might necessitate an individual adaption of the (model) guide that is developed together. Participation in the simulated prescription talks at the end of the course is voluntary and due to time reasons has so far been limited to six simulations per term. Simulations have been standardized by writing a script describing a patient’s history and biography. In brief, the patient has an appointment with his general practitioner for a medication communication regarding drug treatment of a newly diagnosed arterial hypertension. Preexisting bronchial asthma and gout limit first line options according to current treatment guidelines [21]. Thereby students’ preparation of pharmacological subjects and the expenditure of time needed for the conversation itself are reduced. Every simulated talk is restricted to a maximum of 15 minutes. The conversation is observed by two tutors who give feedback on the basis of a checklist (see 2.2.2). The talk is videotaped and the video file is handed over to the student if he or she wants.

The students are asked to fill in portfolios during the course. In three steps participants’ attitudes regarding medication adherence and physician-patient communication in this context are addressed (see Figure 1 (Fig. 1)). Furthermore, students are asked to reflect their own strengths and weaknesses in communication and thereby to define own goals for this course (portfolio task 2). In terms of a pre-post reflection, students are invited to state one to two weeks after the course what they think they have picked up finally (portfolio task 3). Portfolio forms are provided online and students are asked to send each task to one of the tutors to allow for individual consideration (e.g. during feedback on the simulated conversation). The elective closes with a short (10-15 minutes) written test and an informal feedback session. 

##### 2.2.2. Model guide for a prescription talk

The content of the (model) conversation guide that is developed together with the students covers current literature findings on drug treatment [e.g. [4], [11], [21], [22] and research on health services and communication [e.g. [7], [8], [12], [15], [23], [24]. Thus, aspects of drug information, risk communication and patient participation are considered. Regarding relevance, correctness and applicability, a first draft of the guide was reviewed by general practitioners associated with our medical faculty. Based upon their feedback slight modifications were made.

There have been three reasons to derive a checklist from our (model) guide on medication communication:

1. The checklist should allow for a specific “semi-quantitative” feedback regarding the simulated conversations at the end of the course.
2. The checklist should be used to test for the applicability of our conversation guide in general.
3. The checklist should allow to assess conversations in other contexts but our elective, too (e.g. conversations conducted by students who did not attend our course).

**Figure 2:**
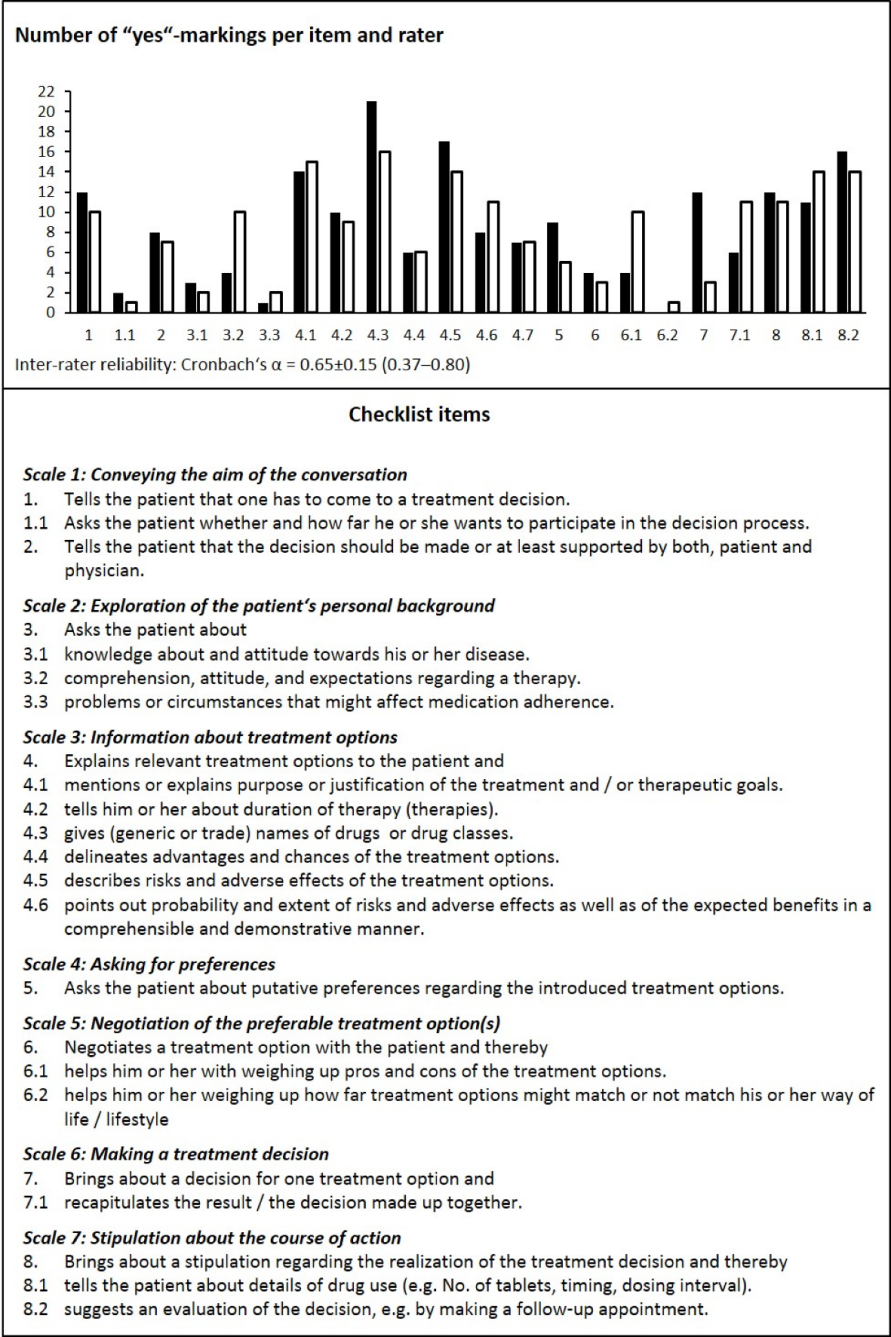
Checklist for evaluating a prescription talk (marking of single items with “yes”, “in parts”, or “no”). Number of “yes”-markings per item by two raters are given who independently assessed 22 simulated conversations.

By using our checklist, we assessed whether and to what extent the several items are realized in a conversation. We chose an ordinal scale (yes/in parts/no) and tested its applicability. The 22 checklist items can be grouped into seven scales (cp. Figure 2) that can be referred to within an individual feedback.

##### 2.2.3. Statistics and analyses

Mean values and standard deviations are given, in case of evaluation data weighed averages. Quality criteria of our checklist were analyzed using SPSS 23 and Excel 2013. As a measure of internal consistency and inter-rater reliability Cronbach’s α was calculated. An α≥0.5 was taken as appropriate for scientific purposes, an α≥0.8 as sufficient for using the items or scales for summative assessment. Items and scales were defined as selective if the coefficient of an item-total correlation was ≥0.32. Descriptive analysis of student conversations by our checklist was based upon frequencies of fully fulfilling a particular item (i.e. number of “yes”-markings). By summarizing content analysis according to Mayring [25] portfolio tasks 2 and 3 were analyzed. For this, the software MAXQDA was used.

## 3. Evaluation / results

### 3.1. Feasibility and acceptance of our elective

Until submission of the manuscript, our elective has been conducted five times. Since the start during winter term 2013/2014 the number of participants has been continuously rising up to 10 on average. After the first piloting phase (2013/2014) slight changes regarding the schedule were made: instead of the input on antihypertensive treatment the first PbL session was chosen as the very starting point to emphasize the student-centered character of the whole elective and thus to avoid wrong expectations in this regard. This schedule (see Figure 1 (Fig. 1)) has proven its worth until now.

The faculty’s course evaluation via the online platform uk-online used German school grades, i.e. “1” as the best and “5” as the worst possible grade. Referring to our elective students’ evaluation during the period 2013-2016 was 1.3±0.2 regarding “lecture”, 1.4±0.2 regarding “small-group teaching”, and 1.6±0.5 regarding “assessment” (i.e. simulation and knowledge test in case of our elective). Written commentaries added by the students appreciated the diversity of learning / teaching formats and the opportunity to apply the course content in a simulated conversation. Some participants claimed more those opportunities during their medical studies and/or involvement of more patient-sided disciplines.

#### 3.2. Checklist to evaluate a medication conversation

The checklist (see Figure 2 (Fig. 2)) was applied by KH and JM to all 22 conversations simulated until winter term 2015/2016. KH and JM judged the applicability of the checklist as good. The ordinal scale allowed for a differentiated assessment of the students’ performance. The checklist was perceived as useful for giving a meaningful feedback. Internal consistency of the checklist as applied by the two raters was α=0.84 and 0.75, respectively. As a measure of inter-rater reliability a Cronbach’s α of 0.52±0.28 for single items was calculated, with rather low values regarding the “in parts”-option. Referring to the scales Cronbach’s α was 0.65±0.15 on average, with three scales out of seven showing an α≥0.75, and another two showing an α>0.6. 13 out of 22 single items turned out to be selective. Referring to the particular sum score all seven scales were selective with correlation coefficients of 0.49±0.11 on average. In summary, quality criteria thus allow to use the checklist for scientific purposes.

#### 3.3. Learning progress and domains of learning goals

For assessing the progress of learning and the development of competencies we addressed cognitive and affective domains via checklist and portfolio, respectively [cp. [26]].

##### 3.3.1. Cognitive domain of learning: application of the conversation guide 

Demonstration of competencies covered by our (model) conversation guide in simulated prescription talks was assessed by using our checklist (see Figure 2 (Fig. 2)). Deficits regarding application of some steps of SDM were revealed, e.g. initiation of patient participation at the beginning of the conversation (scale #1). The desire for participation was inquired by only two (rater 1) or even one (rater 2) of 22 students. Inquiry of individual circumstances that might affect the course of a treatment (item #3.3 of scale #2) was sufficient (i.e. marked with “yes”) in only one (rater 1) or two (rater 2) conversations. Furthermore, weighing up treatment options together with the patient and thereby taking into account patient’s life style and personal situation (item #6.2) was realized once (rater 2) or not at all (rater 1). Of note, students were free to decide whether and to what extent they applied the (model) guide. Thus, lack of a particular issue is not inevitably indicating a failure but may be due to a willful decision. However, only two students frankly stated that they disliked the guide relating to their own conversations.

Other aspects of a prescription talk were realized more often. For example, in many simulated conversations description of the different treatment options (scale #3) comprised explanations of several drug-related information: items #4.1, #4.3 and #4.5 were marked with “yes” by rater 1 or 2 in 63% (14) or 68% (15), 95% (21) or 72% (16), and 77% (17) or 63% (14) of the conversations, respectively. Conversations were closed according to scale #7 (“bringing about a stipulation regarding the realization of the treatment decision”) in 50% (item #8) and 63% (items #8.1 and #8.2) of the cases.

##### 3.3.2. Affective domain of learning: attitude and motivation

The opportunity to record attitudes and the individual learning progress by answering the portfolio questions 2 and 3 (see Table 1 (Tab. 1)) was taken by 28 and 18 out of 40 participants, respectively. 13 out of 18 students stated that an initial uncertainty regarding the prescription talk was abolished during the course and that eventually there was a good starting point for future medication communication (additional practical training assumed). Answers on the question “What did I pick up from this course?” have been merged into categories (see Table 1 (Tab. 1)). Overall, an enhanced awareness of the impact and potential of physicians’ communication in terms of communicating purposefully and strengthening patients’ self-determination by explaining and engaging was indicated.

## 4. Discussion

During the last years, communication competencies became more and more important in medical education as can be taken e.g. from the German nationwide catalogue of learning objectives (NKLM, http://www.nklm.de) from 2015 [27]. Prior to this, education and research focused rather on history taking since this plays an important role in initializing physician-patient communication. However, problems evolving later on, e.g. when a treatment decision has to be made, have not yet been considered sufficiently. In this context Elwyn et al. complained a “neglected second half of the consultation” [15]. In spite of efforts to fight research deficits regarding this “second half” [6], [8], [15], [28], [29], [30], [31], [32], there is still a gap, namely the prescription talk as a common endpoint of the consultation. Furthermore, the obvious problems with poor medication adherence [4], [33] and the putative importance of the prescription talk for enhancing adherence [34], [35] necessitates emphasizing “the second half of the consultation” in (undergraduate) medical education from our point of view. According to a recent review, the use of simulation patients is promising particularly in pharmacological education [26], since this would not only enhance students' sense of responsibility regarding drug safety, but also foster a patient-oriented communication. In agreement with this, the portfolio entries of our participants show that the offered syllabus regarding medication communication in general and the simulation of such a conversation in particular enhance the awareness of patients’ desire to participate. In addition, many of our students stated that the simulated prescription talk was an important and helpful experience and in our opinion, it is the indispensable highlight of our elective.

The project described here may play an important role to fulfil requirements resulting from the above-mentioned NKLM in the area of negotiation (chapter #14c of the NKLM). Examples directly related to clinical pharmacology and pharmacotherapy are competencies and learning goals aiming at adequate risk communication (#14c.4.2), addressing non-adherence (#14c.4.1.1), or applying the method of shared decision-making (#14c.2.1.9).

Given that our elective in the following proves its worth, we will integrate this course into the obligatory part of our medical curriculum. Furthermore, a transfer to disciplines characterized by extensive drug prescription (e.g. general medicine or internal medicine) is planned, both in under- and postgraduate education. A current study aims at proving applicability of our (model) conversation guide in physicians’ daily routine and putative effects on satisfaction with a prescription talk in a clinical setting.

Limitations of our study are the rather low numbers of participants. Furthermore, we have to assume a selection bias since it is likely that mainly students with a particular interest in the treated subject have chosen this elective. Of note, the University of Cologne runs a reformed medical curriculum. Taken together the transferability of our results thus might be limited. It should be considered that pharmacologists in Cologne are not directly involved in patient care and that we thus mainly refer to literature data and less or more informal feedback from clinical colleagues. It is particularly important to note that our (model) guide for medication communication is not yet clinically validated, though it covers actual deficits of advanced medical students [36].

## 5. Conclusions

After attending our newly developed elective medical students feel more secure regarding prescription talks and state an enhanced awareness of not always obvious but definitely relevant aspects like patient participation and adherence improvement. Though the realization of specific competencies has to be practiced further, our participants’ feedback indicates that we were successful in pointing out new perspectives on physician-patient communication and an adequate, if possible pari passu engagement of patients.

## Funding

This work was supported financially by the rectorate of the University of Cologne [Inno-2013-3-3].

## Competing interests

The authors declare that they have no competing interests.

## Figures and Tables

**Table 1:**
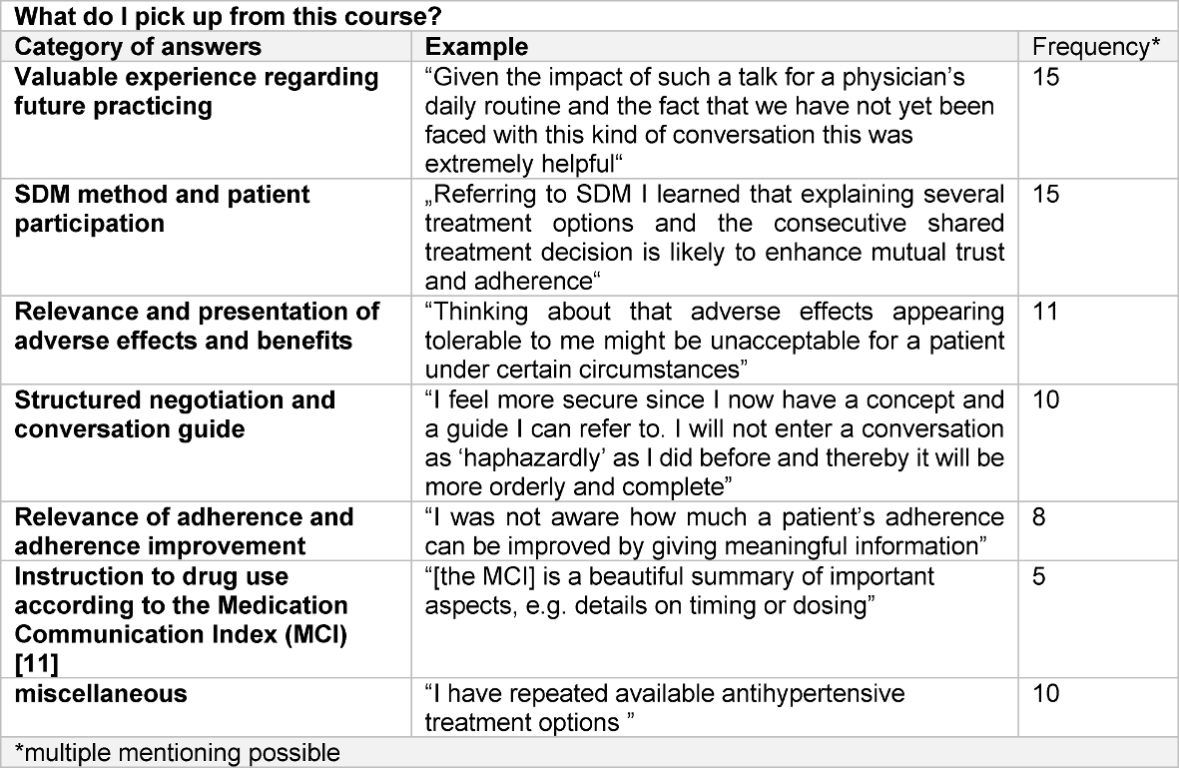
Results of the analysis of students’ portfolio entries regarding the question “What did I pick up from this course?”. Using the summarizing content analysis according to Mayring [25] individual answers were merged into categories (first column). For each category, an exemplary portfolio entry is provided (second column). The third column gives the frequency of portfolio entries covering a particular category.

## References

[1] Richard C, Lussier M-T (2006). Nature and frequency of exchanges on medications during primary care encounters. Patient Educ Couns.

[2] Stevenson FA, Barry CA, Britten N, Barber N, Bradley CP (2000). Doctor–patient communication about drugs: the evidence for shared decision making. Soc Sci Med.

[3] Sabaté E (2003). Adherence to long-term therapies: evidence for action.

[4] Matthes J, Albus C (2014). Improving adherence with medication: a selective literature review based on the example of hypertension treatment. Dtsch Arztebl Int.

[5] Simpson SH, Eurich DT, Majumdar SR, Padwal RS, Tsuyuki RT, Varney J, Johnson JA (2006). A meta-analysis of the association between adherence to drug therapy and mortality. BMJ.

[6] Karnieli-Miller O, Eisikovits Z (2009). Physician as partner or salesman? Shared decision-making in real-time encounters. Soc Sci Med.

[7] Loh A, Simon D, Kriston L, Härter M (2007). Patientenbeteiligung bei medizinischen Entscheidungen. Dtsch Arztebl.

[8] Cullati S, Courvoisier DS, Charvet-Bérard AI, Perneger TV (2011). Desire for autonomy in health care decisions: a general population survey. Patient Educ Couns.

[9] Guadagnoli E, Ward P (1998). Patient participation in decision-making. Soc Sci Med.

[10] Makoul G, Arntson P, Schofield T (1995). Health Promotion in Primary Care: Physician-Patient Communication and Decision Making About Prescription Medications. Soc Sci Med.

[11] Tarn DM, Heritage J, Paterniti DA, Hays RD, Kravitz RL, Wenger NS (2006). Physician Communication When Prescribing New Medications. Arch Intern Med.

[12] Barry CA, Bradley CP, Britten N, Stevenson FA, Barber N (2000). Patients' Unvoiced Agendas in General Practice Consultations: Qualitative Study. BMJ.

[13] Ziegler DK, Mosier MC, Buenaver M, Okuyemi K (2001). How Much Information About Adverse Effects of Medication Do Patients Want from Physicians?. Arch Intern Med.

[14] Klok T, Kaptein AA, Brand PL (2015). Non-adherence in children with asthma reviewed: The need for improvement of asthma care and medical education. Pediatr Allergy Immunol.

[15] Elwyn G, Edwards A, Kinnersley P (1999). Shared decision-making in primary care: the neglected second half of the consultation. Br J Gen Pract.

[16] Dearden E, Mellanby E, Cameron H, Harden J (2015). Which non-technical skills do junior doctors require to prescribe safely? A systematic review. Br J Clin Pharmacol.

[17] Hauser K, Koerfer A, Kuhr K, Albus C, Herzig S, Matthes J (2015). Outcome-Relevant Effects of Shared Decision Making. Dtsch Arztebl Int.

[18] Joosten EA, DeFuentes-Merillas L, De Weert G, Sensky T, Van Der Staak C, de Jong CA (2008). Systematic review of the effects of shared decision-making on patient satisfaction, treatment adherence and health status. Psychother Psychosom.

[19] Charles C, Gafni A, Whelan T (1997). Shared decision-making in the medical encounter: what does it mean?(or it takes at least two to tango). Soc Sci Med.

[20] Charles C, Gafni A, Whelan T (1999). Decision-making in the physician-patient encounter: revisiting the shared treatment decision-making model. Soc Sci Med.

[21] Mancia G, Fagard R, Narkiewicz K, Redon J, Zanchetti A, Böhm M, Christiaens T, Cifkova R, De Backer G, Dominiczak A, Galderisi M, Grobbee DE, Jaarsma T, Kirchhof P, Kjeldsen SE, Laurent S, Manolis AJ, Nilsson PM, Ruilope LM, Schmieder RE, Sirnes PA, Sleight P, Viigimaa M, Waeber B, Zannad F, Redon J, Dominiczak A, Narkiewicz K, Nilsson PM, Burnier M, Viigimaa M, Ambrosioni E, Caufield M, Coca A, Olsen MH, Schmieder RE, Tsioufis C, van de Borne P, Zamorano JL, Achenbach S, Baumgartner H, Bax JJ, Bueno H, Dean V, Deaton C, Erol C, Fagard R, Ferrari R, Hasdai D, Hoes AW, Kirchhof P, Knuuti J, Kolh P, Lancellotti P, Linhart A, Nihoyannopoulos P, Piepoli MF, Ponikowski P, Sirnes PA, Tamargo JL, Tendera M, Torbicki A, Wijns W, Windecker S, Clement DL, Coca A, Gillebert TC, Tendera M, Rosei EA, Ambrosioni E, Anker SD, Bauersachs J, Hitij JB, Caulfield M, De Buyzere M, De Geest S, Derumeaux GA, Erdine S, Farsang C, Funck-Brentano C, Gerc V, Germano G, Gielen S, Haller H, Hoes AW, Jordan J, Kahan T, Komajda M, Lovic D, Mahrholdt H, Olsen MH, Ostergren J, Parati G, Perk J, Polonia J, Popescu BA, Reiner Z, Rydén L, Sirenko Y, Stanton A, Struijker-Boudier H, Tsioufis C, van de Borne P, Vlachopoulos C, Volpe M, Wood DA (2013). 2013 ESH/ESC guidelines for the management of arterial hypertension: the Task Force for the Management of Arterial Hypertension of the European Society of Hypertension (ESH) and of the European Society of Cardiology (ESC). Eur Heart J.

[22] Schott G, Berthold H (2005). Pharmakovigilanz: Empfehlungen zur Meldung unerwünschter Arzneimittelwirkungen durch die Ärzteschaft. ZFA.

[23] Gigerenzer G, Gaissmaier W, Kurz-Milcke E, Schwartz LM, Woloshin S (2007). Helping doctors and patients make sense of health statistics. Psychol Sci Public Interest.

[24] Osterberg L, Blaschke T (2005). Adherence to medication. New Engl J Med.

[25] Mayring P (2015). Qualitative Inhaltsanalyse. Grundlagen und Techniken.

[26] Aura SM, Sormunen MS, Jordan SE, Tossavainen KA, Turunen HE (2015). Learning Outcomes Associated With Patient Simulation Method in Pharmacotherapy Education: An Integrative Review. Simul Healthc.

[27] Fischer MR, Bauer D, Mohn K, NKLM Projektgruppe (2015). Endlich fertig! Nationale Kompetenzbasierte Lernzielkataloge Medizin (NKLM) und Zahnmedizin (NKLZ) gehen in die Erprobung. GMS Z Med Ausbild.

[28] Elwyn G, Edwards A, Rhydderch M, Härter M, Loh A, Spiess C (2005). Shared Decision Making: das Konzept und seine Anwendung in der klinischen Praxis. Gemeinsam Entscheiden—Erfolgreich Behandeln Neue Wege für Arzte und Patienten Im Gesundheitswesen.

[29] Klemperer D, Härter M, Loh A, Spiess C (2005). Partizipative Entscheidungsfindung in Deutschland–Handlungsfelder zur Verbesserung der Entscheidungsqualität. Gemeinsam entscheiden–erfolgreich behandeln.

[30] Pollock K (2005). Concordance in medical consultations: a critical review.

[31] Smith A, Juraskova I, Butow P, Miguel C, Lopez AL, Chang S, Brown R, Bernhard J (2011). Sharing vs. caring - the relative impact of sharing decisions versus managing emotions on patient outcomes. Patient Educ Couns.

[32] Koerfer A, Albus C, Spranz-Fogasy T, Busch A (2015). Dialogische Entscheidungsfindung zwischen Arzt und Patient. Handbuch Sprache in der Medizin.

[33] Bosworth HB, Granger BB, Mendys P, Brindis R, Burkholder R, Czajkowski SM, Daniel JG, Ekman I, Ho M, Johnson M, Kimmel SE, Liu LZ, Musaus J, Shrank WH, Whalley Buono E, Weiss K, Granger CB (2011). Medication adherence: a call for action. Am Heart J.

[34] Albus C, Matthes J (2014). Interventions to enhance adherence to medication. MMW Fortschr Med.

[35] Hauser K, Matthes J, Heiß HW (2016). Medikamentöse Adhärenz. Altersmedizin aktuell.

[36] Hauser K, Matthes J (2017). Medical students' medication communication skills regarding drug prescription-a qualitative analysis of simulated physician-patient consultations. Eur J Clin Pharmacol.

